# Enterovirus detection in different regions of Madagascar reveals a higher abundance of enteroviruses of species C in areas where several outbreaks of vaccine-derived polioviruses occurred

**DOI:** 10.1186/s12879-022-07826-0

**Published:** 2022-11-08

**Authors:** Richter Razafindratsimandresy, Marie-Line Joffret, Soa Fy Andriamandimby, Seta Andriamamonjy, Sendraharimanana Rabemanantsoa, Vincent Richard, Francis Delpeyroux, Jean-Michel Heraud, Maël Bessaud

**Affiliations:** 1 Virology Unit, Institut Pasteur de Madagascar, 101 Antananarivo, Madagascar; 2 Institut Pasteur, 75015 Paris, France; 3 International Affairs Department, Institut Pasteur, 75015 Paris, France

**Keywords:** Human enterovirus, Genotype, Madagascar, Vaccine-derived poliovirus

## Abstract

**Background:**

Poliomyelitis outbreaks due to pathogenic vaccine-derived polioviruses (VDPVs) are threatening and complicating the global polio eradication initiative. Most of these VDPVs are genetic recombinants with non-polio enteroviruses (NPEVs) of species C. Little is known about factors favoring this genetic macroevolution process. Since 2001, Madagascar has experienced several outbreaks of poliomyelitis due to VDPVs, and most of VDPVs were isolated in the south of the island. The current study explored some of the viral factors that can promote and explain the emergence of recombinant VDPVs in Madagascar.

**Methods:**

Between May to August 2011, we collected stools from healthy children living in two southern and two northern regions of Madagascar. Virus isolation was done in RD, HEp-2c, and L20B cell lines, and enteroviruses were detected using a wide-spectrum 5ʹ-untranslated region RT-PCR assay. NPEVs were then sequenced for the VP1 gene used for viral genotyping.

**Results:**

Overall, we collected 1309 stools, of which 351 NPEVs (26.8%) were identified. Sequencing revealed 33 types of viruses belonging to three different species: Enterovirus A (8.5%), Enterovirus B (EV-B, 40.2%), and Enterovirus C (EV-C, 51.3%). EV-C species included coxsackievirus A13, A17, and A20 previously described as putative recombination partners for poliovirus vaccine strains. Interestingly, the isolation rate was higher among stools originating from the South (30.3% vs. 23.6%, p-value = 0.009). EV-C were predominant in southern sites (65.7%) while EV-B predominated in northern sites (54.9%). The factors that explain the relative abundance of EV-C in the South are still unknown.

**Conclusions:**

Whatever its causes, the relative abundance of EV-C in the South of Madagascar may have promoted the infections of children by EV-C, including the PV vaccine strains, and have favored the recombination events between PVs and NPEVs in co-infected children, thus leading to the recurrent emergence of recombinant VDPVs in this region of Madagascar.

**Supplementary Information:**

The online version contains supplementary material available at 10.1186/s12879-022-07826-0.

## Background

Members of the species *Enterovirus C* (EV-C, genus *Enterovirus*, family *Picornaviridae*), the three serotypes of poliovirus (PVs) are the causal agent of poliomyelitis, an infectious disease characterized by acute flaccid paralysis. Launched in 1988 and coordinated by the World Health Organization, the Global Polio Eradication Initiative (GPEI) aims to eradicate the PVs. One GPEI pillar is mass vaccination campaigns that target children under 5 years old. Two efficient vaccines were developed in the 1950s–1960s: the injectable polio vaccine, which contains inactivated particles of each of the three serotypes and the oral polio vaccine (OPV), which contains three attenuated strains. When orally administrated, these strains replicate in the gut of the vaccine recipient and induce a strong mucosal immunity that limits the replication of PVs during subsequent infections with PVs [1, 2]. Therefore, OPV effectively block PV circulation in humans. OPV has been the main tool used by the GPEI, which succeeded in eliminating wild PV-2 and -3 while the area of circulation of wild PV-1 strains has been restricted to two countries only, Pakistan and Afghanistan.

Nonetheless, a low vaccine coverage can enable the circulation of OPV strains in non-vaccinated children. Long transmission chains allow the genetic drift of the vaccine strains, which can lose their attenuation determinants and recover a pathogenic phenotype similar to that of wild strains. Polio outbreaks due to these circulating vaccine-derived polioviruses (cVDPVs) have been regularly reported since 2000 in areas where the vaccine coverage is low [3]. These cVDPVs are threatening the benefit of the vaccination campaigns and complexifying the GPEI strategies.

Most cVDPVs detected worldwide feature recombinant genomes made of genetic sequences originating from the OPV strains and from non-polio enteroviruses (NPEVs). All cVDPVs retain the PV vaccine capsid-encoding region but large parts of the other regions of their genomes come from NPEVs [4]. In particular, CVA17 and -A20 and to some extent CVA11 and -A13 appeared to be possible recombinant partners of polioviruses.

The main factor that favors the emergence of pathogenic cVDPVs is the low vaccine coverage that allows the establishment of long transmission chains between non-immunized people. Nonetheless, it is possible that other factors promote the emergence and the circulation of cVDPVs. In particular, it is likely that recombination events between the PV vaccine strains and co-circulating EV-Cs accelerate the reversion of their attenuated phenotype.

In the 2000s and 2010s, Madagascar experienced several independent emergences of cVDPVs, of which some were responsible of poliomyelitis cases [5, 6]. All these cVDPVs featured recombinant genomes. Noteworthy, most of them were detected in three southern regions of the island. The 2001–2002 poliomyelitis outbreak took place in two Southern provinces of Madagascar, Toliara and Taolagnaro [6]. Three years later, in 2005, another outbreak occurred in Toliara province [5]. In 2013, a study in the same province highlighted the circulation of different lineages of VDPVs, although no poliomyelitis cases were linked to these viruses [7]. These regions do not have peculiar high population density that could explain the emergence of cVDPVs [8]. Huge differences regarding the vaccine coverage does not seem to explain either why the cVDPVs preferentially emerges in the southern part of the island.

Many other factors could explain discrepancy between the southern and northern regions regarding the frequency of cVDPVs emergence: climate, access to potable water, eating habits, etc. One factor could be differences in the NPEV populations circulating in children. To address this hypothesis, we compared the NPEV populations isolated from children stools collected in the meantime in four regions of Madagascar: Toliara and Taolagnaro in the South, and Mahajanga and Antseranana in the North.

## Methods

### Sites selection

**Fig. 1:**
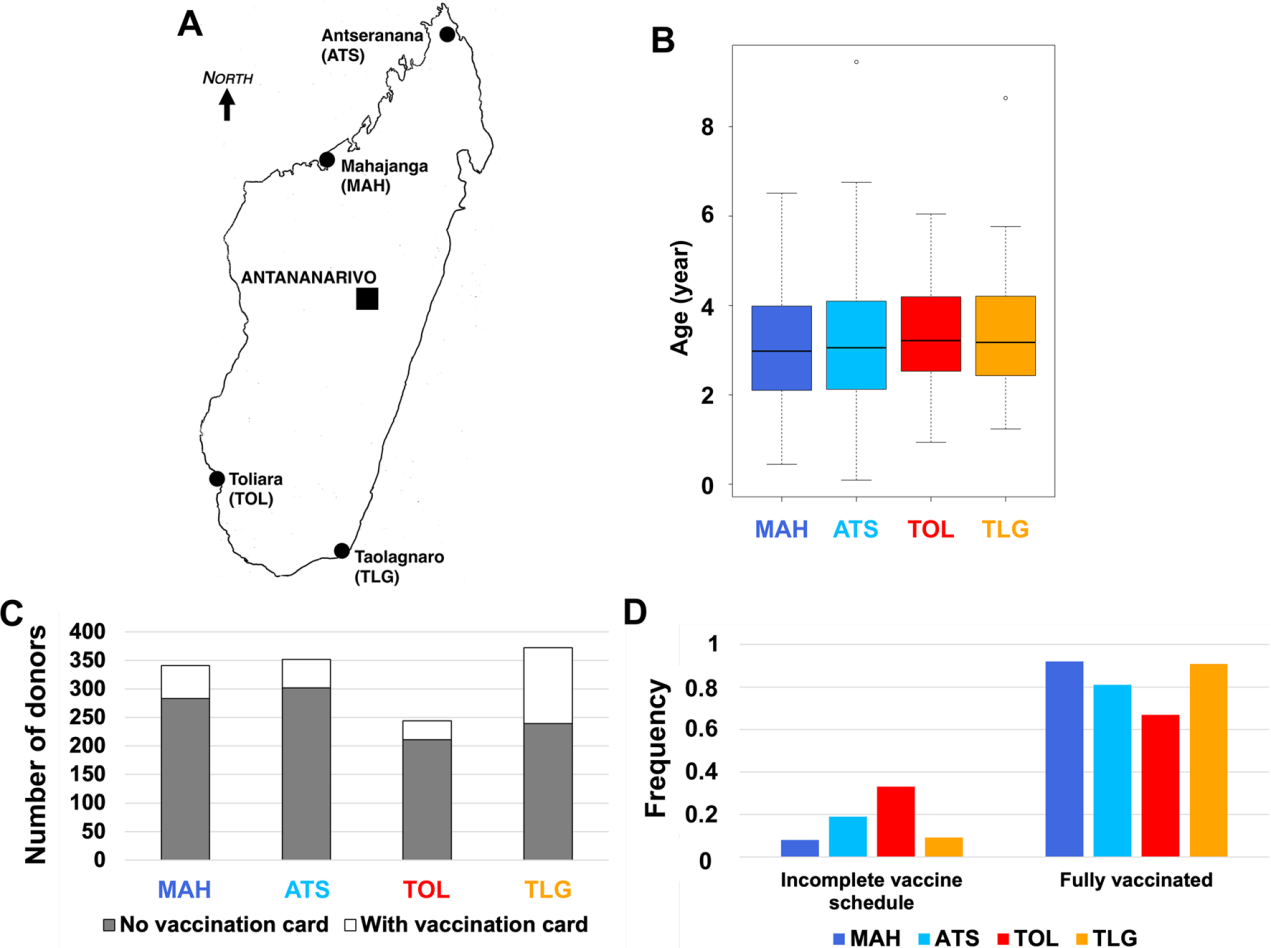
**A** Map of Madagascar. Antananarivo, the capital city, and the four capitals of the districts in which the samples were collected are indicated. **B** Boxplot of the age of the donors in each district. **C** Number of donors with and without vaccination card in each district. **D** Proportion of donors fully vaccinated amongst the donors with vaccination card in each district

We compared the data from the southern sites (Toliara (TOL) and Taolagnaro (TLG)) and the data from the northern sites (Mahajanga (MAH) and Antseranana (ATS)) in which no VDPV outbreak was reported before 2011 (Fig. 1A). The difference of fecal carriages or diarrhea etiologies between the North and South regions of Madagascar have been previously reported [9] and have driven the choice of these two different climatic regions. Socio-economic overall status, climate, as well as vaccine coverage were rather similar during the collection period. In 2011, the OPV coverage rates were 66.1% and 78.5% in the TOL and TLG region (located in the south of the island where most cVDPVs emerged) respectively, and 67.5% and 80.1% in MAH and ATS regions (located in the north of the island) respectively.

### Field investigations and samples collection

Four field missions were performed in May and June 2011 in the South, and in July and August 2011 in the North. Stool specimens were collected from healthy children under 5 years old randomly selected in the four selected sites. A questionnaire including data on date of birth, sex, site of enrolment, previous routine immunization (based on health card), and immunization campaign with OPV (based on parent’s declarations) was completed for each child.

### Cell lines and virus isolation

RD (human rhabdomyosarcoma), HEp-2c (human cervix carcinoma, HeLa derivatives), and L20B (mouse fibroblasts expressing the PV human receptor CD155) cell lines were used in this study. L20B, a genetically engineered mouse cell line, is intended for the specific isolation of PV but it has been reported that it can also propagate some *Enterovirus A* and *D* (EV-A, -D) [10, 11]. All cell lines were grown as monolayers in Dulbecco’s Modified Eagle Medium (D-MEM) supplemented with 5% (RD and HEp-2c) or 10% (L20B) fetal calf serum. Stool specimens were treated with chloroform and used to inoculate the cells, following the standard procedures described in the laboratory manual for the WHO Global Polio Laboratory Network [12]. Inoculated monolayers were microscopically checked for 5 days to detect the appearance of cytopathogenic effects (CPE). To increase the sensitivity of virus isolation, a blind passage (inoculation in the same cell line) was carried out using the supernatant of the inoculated cultures which had remained negative (no CPE), and newly inoculated cells were then checked for the next 5 days. All viruses isolated from RD and HEp-2c cells were systematically inoculated onto L20B cells, and the resulting poliovirus isolates were further typed. The isolates showing CPE only on RD or HEp-2c but not on L20B cells were considered to be NPEVs and were further typed by molecular methods (see below).

### Poliovirus intratypic differentiation

To determine whether the PVs isolates were from vaccine strain or vaccine-derived strain or wild strain, the standard Intratypic Detection assay was used [13]. It consists of parallel real-time RT-PCRs (rRT-PCR) that target the VP1-encoding region.

### RNA extraction and gene amplification

Viral RNA was extracted from 140 µL of infected cell culture supernatants using a QIAamp viral RNA mini kit (Qiagen, France) according to the manufacturer’s instructions. The RNA extract was eluted in 65 µL of RNase-free water (Sigma-Aldrich, France). The random hexamer p(dN)6 primer (Roche Diagnostics, Germany) was used for the reverse transcription of the viral RNA into cDNA, which was then amplified by PCR using the EV2/EV3 primers (targeting a portion of the most conserved 5′UTR of the EV genome), as described previously [14]. All samples positive in 5′UTR were subjected to RT-PCR with primer pairs previously described to amplify the VP1-encoding region [15, 16]. Amplicons were analyzed by agarose gel electrophoresis. If multiple bands were present in the gel, the amplicons were purified with a QIAquick gel extraction kit (Qiagen, France) according to the manufacturer’s protocol.

### VP1 nucleotide sequencing and sequence analyses

Amplified products were sent for sequencing to the Beckman Coulter Genomics Company (Genewiz Company, now Takley, UK). Sequencing was performed in both directions (forward and reverse) with the primer couple used for gene amplification. The consensus sequences of each gene amplified for every isolate obtained with the forward and reverse primers were verified and corrected, using CEQ 2000XL version 4.0.0 software (Villepinte, France) or CodonCode Aligner (Centerville, MA).

### Molecular typing of NPEVs isolates

Nucleotide sequences corresponding to the partial (3′ one-third or 5′ one-half) or complete VP1 capsid gene for every NPEV isolate were pairwise compared with the homologous sequences of prototype strains retrieved from sequences databases, as previously reported [17, 18]. In most cases, nucleotide identities with the corresponding prototype strains were equal to or higher than the type assignment nucleotide and amino acid thresholds (≥ 75 and ≥ 85% for all human enteroviruses respectively; excepting EV-C ≥ 88% in amino acid) [17].

### Full-length genome sequencing and phylogenetic analyses

EV-C genomes were sequenced through Illumina sequencing following a technique already reported [19]. The complete VP1 nucleotide sequences were aligned using the ClustalW program and phylogenetic trees were reconstructed using the MEGA X program [20]. The evolutionary history was inferred by using the Maximum Likelihood method and Tamura-Nei model. The trees are drawn to scale, with branch lengths measured in the number of substitutions per site. Sequences of interest previously reported were retrieved from GenBank (Additional file 1: Table S1).

### Statistical analyses

Statistical analyses were performed using R software (http://www.R-project.org/). The statistical differences of North/South ratio between different human enteroviruses types identified were tested by Fisher’s exact test.

## Results

### Stools specimens collection

Overall, 1309 stool specimens were collected in 2011. Geographically, 616 specimens came from southern sites (244 and 372 from TOL and TLG, respectively) and 693 from northern sites (341 and 352 from MAH and ATS, respectively). The sex ratio (F:M) among the donors was 0.93. Age ranged from 1 month to 9.4 years with a median of 3.1 years. The age distribution was similar in all sites (Fig. 1B). Polio vaccination records were unavailable for the vast majority (1035 out of 1309) of the donors (Fig. 1C). Among the donors, whose polio vaccine status could be established by immunization cards, most were fully vaccinated (Fig. 1D), although the proportion of donors fully vaccinated in TOL (67%) was notably lower than the ones observed in other regions, which all exceeded 80%.

### Virus isolation

**Fig. 2:**
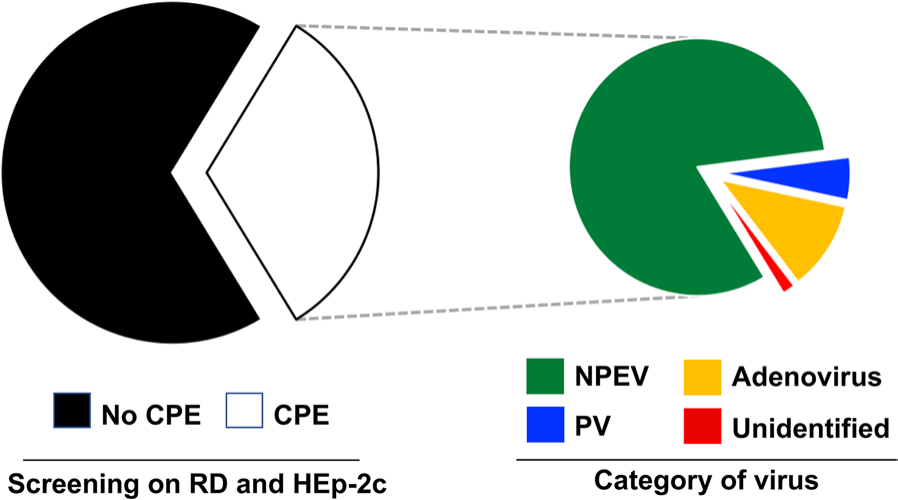
Detection of viruses through screening on cell cultures and identification by molecular assays

Of the 1309 specimens, 426 (32.5%) induced CPE in cell cultures (Fig. 2), of which only 23 (5.4%) were positive on L20B cells, and were identified as PVs. Of the 403 remaining stool extracts, 55 were not molecularly identified as enteroviruses (not amplifiable in 5′UTR by RT-PCR test) and 48 were subsequently identified as adenoviruses, based on immunofluorescence assay. Seven isolates remained unidentified. Finally, 351 NEPVs were isolated from 348 supernatants (three coinfections were noted). Overall, the global isolation rate was 26.8% (351/1309). The isolation rate was statistically higher among samples originated from the south (30.3%) when compared to the samples collected in the north (23.6%) (p-value = 0.009).

### Molecular typing

From the 23 positive L20B supernatants, the intratypic differentiation rRT-PCR assays identified four PV-1, 12 PV-2, and seven PV-3. Twenty were shown to be closely related to the original Sabin strains (Sabin-like strains, according to the Global Polio Lab Network’s algorithm) and three were identified as VDPVs of type 2. These VDPVs were not linked to any acute flaccid paralysis cases. This finding was previously published by Razafindratsimandresy et al. [7]*.*

The 351 NPEVs were molecularly typed based on their VP1-encoding sequences [17, 18]. They were allocated into 33 types of three *Enterovirus* species: 51.3% (180/351) of the isolates were EV-C, 40.2% (141/351) were EV-B, and 8.5% (30/351) were EV-A. As previously reported, a huge discrepancy was observed between the cell lines regarding their respective sensitivity to NPEVs of species B and C (Additional file 2: Figure S1). Out of 180 EV-C, only 17 were detected on RD cells; by contrast, most EV-B (134/141) gave CPE on RD cells, while only 58 gave CPEs on HEp-2c cells, of which seven were isolated on this cell line only.

**Fig. 3:**
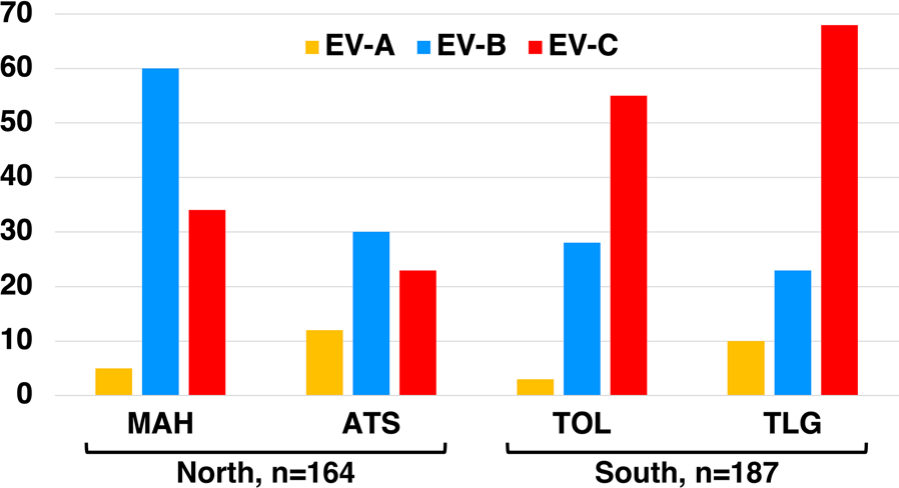
Number of EV-A, -B and -C isolates found in each sampling region

**Fig. 4:**
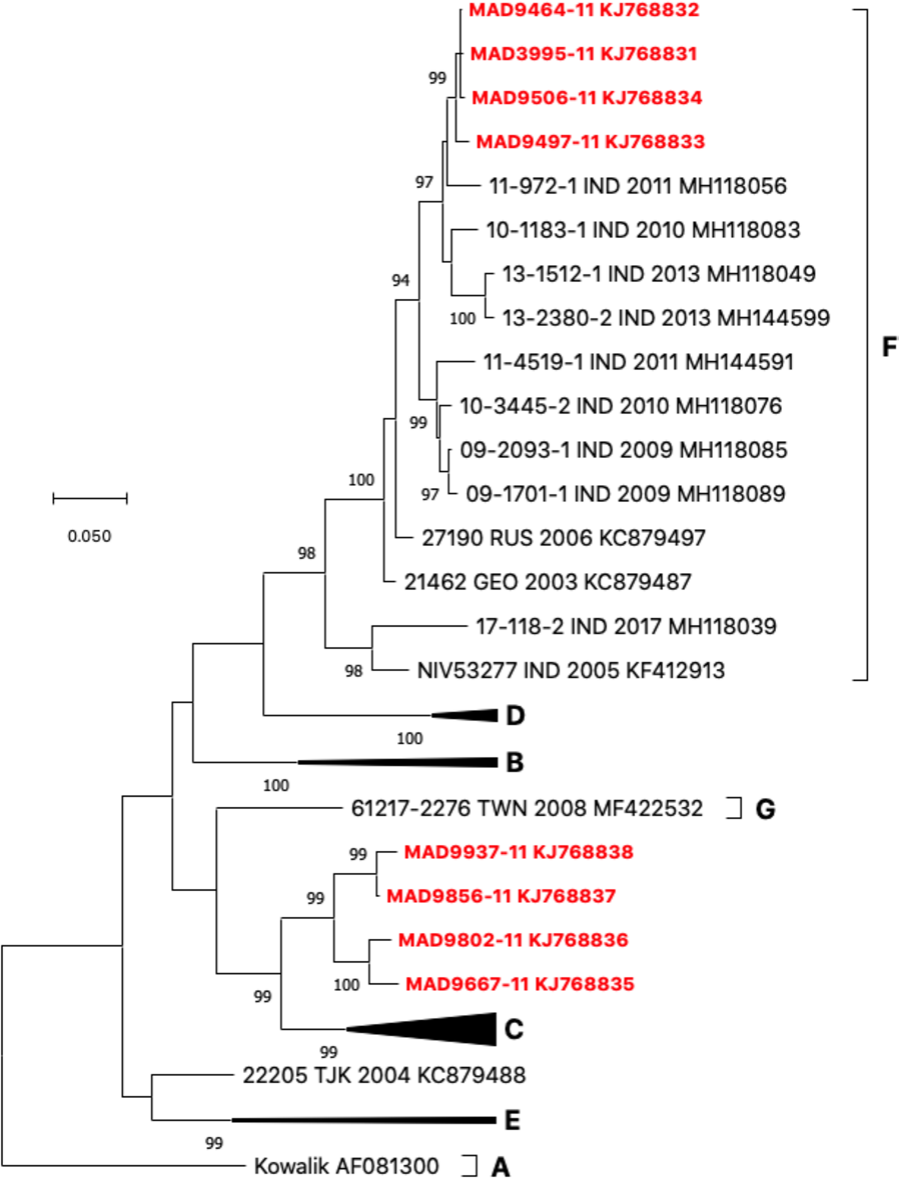
Phylogenetic relationships of CVA10 strains based on the full-length VP1 sequence. The samples from this study are in red with their respective collection site. The other sequences are named by using their respective GenBank accession number, country (ISO 3166-1 alpha-3 codes) and year of sampling, when known; some lineages have been collapsed for better legibility. The percentage of replicate trees in which the associated taxa clustered together in the bootstrap test (1000 replicates) are shown next to the branches; bootstrap values < 95% were hidden

Interestingly, the proportion of EV-B and EV-C among NPEVs substantially differ in the northern and southern parts of the island (Fig. 3). EV-C were predominant in the southern sites, accounting for about two-thirds (123/187) of the viruses isolated in this region, while they accounted for only one-third of the isolates in the northern part (65.7% vs 34.7%, p-value < 10^–8^) (Table 1). By contrast, EV-B were predominant in the northern sites with a rate of 54.9% (90/164). In both regions, only few EV-As were detected and no significant difference was found regarding the EV-A prevalence between the northern and the southern regions. EV-A accounted for 8.5% of the isolates. They were allocated in nine types: CVA2, CVA4, CVA5, CVA6, CVA7, CVA10, CVA14, EV-A71, and EV-A120. CVA7 and CVA10 predominated, accounting for 36.7% and 30.0% of all EV-A, respectively (Table 1). Previous studies classified CVA10 strains into several genotypes based on the VP1-encoding sequence [21, 22]. The four CVA10 found in MAH fell in the genotype F and were close to strains circulating in Indian the 2010–2013 period (Fig. 4). Interestingly, the four isolates from ATS formed together a cluster that did not fall into any described genotype.

**Table 1:** Species and types of the NPEVs isolated according to districts of the study (June–August 2011)

Species/types	Sites				Total n (%)
	MAH n (%)	ATS n (%)	TOL n (%)	TLG n(%)	
CVA7	1 (3.3%)	4 (13.3%)	–	6 (20.0%)	11 (36.7%)
CVA10	4 (13.3%)	5 (16.7%)	–	–	9 (30.0%)
CVA5	–	–	–	3 (10.0%)	3 (10.0%)
CVA6	–	1 (3.3%)	1 (3.3%)	–	2 (6.7%)
CVA2	–	1 (3.3%)	–	–	1 (3.3%)
CVA4	–	1 (3.3%)	–	–	1 (3.3%)
CVA14	–	–	1 (3.3%)	–	1 (3.3%)
EV-A71	–	–	–	1 (3.3%)	1 (3.3%)
EV-A120	-	–	1 (3.3%)	–	1 (3.3%)
***Enterovirus A***	**5 (16.7%)**	**12 (40.0%)**	**3 (10.0%)**	**10 (33.3%)**	**30 (100%)**
CVB4	14 (9.9%)	7 (5.0%)	2 (1.4%)	–	23 (16.3%)
E7	3 (2.1%)	-	3 (2.1%)	10 (7.1%)	16 (11.3%)
CVB2	9 (6.4%)	3 (2.1%)	2 (1.4%)	–	14 (9.9%)
E11	4 (2.8%)	9 (6.4%)	1 (0.7%)	–	14 (9.9%)
E6	4 (2.8%)	–	6 (4.2%)	–	10 (7.1%)
E12	–	–	8 (5.7%)	2 (1.4%)	10 (7.1%)
E13	6 (4.2%)	1 (0.7%)	1 (0.7%)	2 (1.4%)	10 (7.1%)
E20	6 (4.2%)	–	2 (1.4%)	–	8 (5.7%)
E2	5 (3.5%)	–	-	2 (1.4%)	7 (5.0%)
E14	3 (2.1%)	2 (1.4%)	-	–	5 (3.5%)
E24	1 (0.7%)	2 (1.4%)	-	2 (1.4%)	5 (3.5%)
E29	1 (0.7%)	2 (1.4%)	-	2 (1.4%)	5 (3.5%)
E30	1 (0.7%)	2 (1.4%)	2 (1.4%)	–	5 (3.5%)
E1	–	–	–	2 (1.4%)	2 (1.4%)
E21	1 (0.7%)	–	1 (0.7%)	–	2 (1.4%)
CVB3	–	2 (1.4%)	–	–	2 (1.4%)
CVB5	1 (0.7%)	–	–	1 (0.7%)	2 (1.4%)
E19	1 (0.7%)	–	–	–	1 (0.7%)
***Enterovirus B***	**60 (42.5%)**	**30 (21.3%)**	**28 (19.9%)**	**23 (16.3%)**	**141 (100%)**
CVA13	15 (8.3%)	6 (3.3%)	20 (11.1%)	27 (15.0%)	68 (37.8%)
CVA11	1 (0.5%)	1 (0.5%)	7 (3.9%)	29 (16.1%)	38 (21.1%)
EV-C99	7(3.9%)	3 (1.7%)	10 (5.5%)	9 (5.0%)	29 (16.1%)
CVA24	7 (3.9%)	8 (4.4%)	6 (3.3%)	1 (0.5%)	22 (12.2%)
CVA20	4 (2.2%)	5 (2.8%)	6 (3.3%)	2 (1.1%)	17 (9.4%)
CVA17	–	–	6 (3.3%)	–	6 (3.3%)
***Enterovirus C***	**34 (18.9%)**	**23 (12.8%)**	**55 (30.5%)**	**68 (37.8%)**	**180 (100%)**

*CV* Coxsackievirus, *EV* Enterovirus, *E* Echovirus, *MAH* Mahajanga, *ATS* Antseranana, *TOL* Toliara, *TLG* Taolagnaro

The EV-A71 isolate found in this study (MAD3126-11, GenBank accession no. HG421069) was previously described by Bessaud et al. [23]. It constitutes the prototype of a new clade tentatively called “genogroup F” that has never been reported outside Madagascar.

One isolate (MAD2741-11, GenBank accession no. KF700245) belonged to the type EV-A120. It was the second isolate of this type, after the first detection of an EV-A120 in Papua New Guinea in 2009. Since, EV-A120s have been described in Trinidad and Tobago, South-Africa, France, Senegal, Pakistan and Tajikistan.

**Fig. 5:**
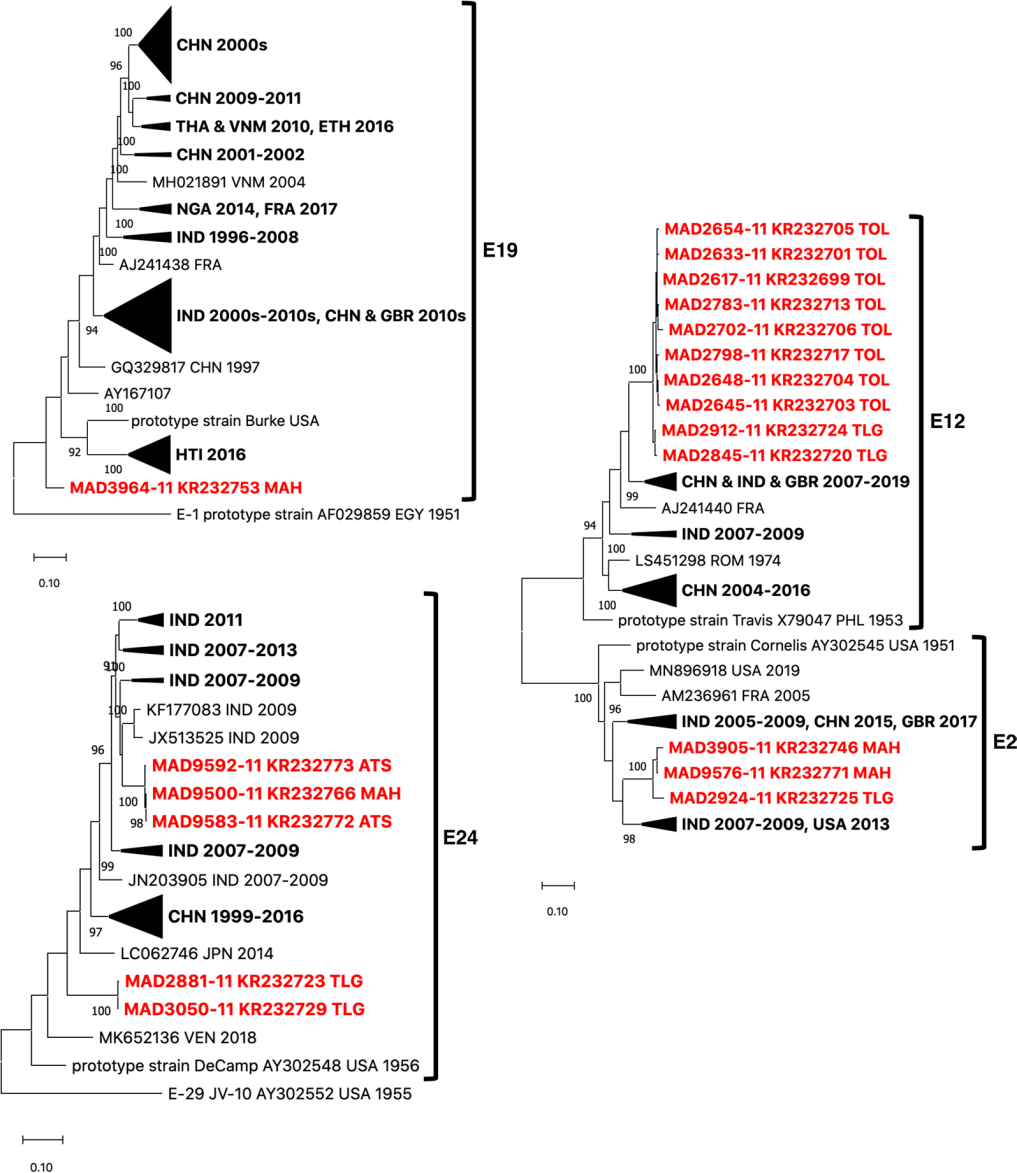
Evolutionary relationships based on the full-length VP1 sequences belonging to the types E19, E2, E12 and E24. The samples from this study are in red with their respective collection site. The other sequences are named by using their respective GenBank accession number, country (ISO 3166-1 alpha-3 codes) and year of sampling, when known; some lineages have been collapsed for better legibility. The evolutionary history was inferred in MEGA X by using the Maximum Likelihood method and Tamura-Nei model. Initial tree(s) for the heuristic search were obtained automatically by applying Neighbor-Join and BioNJ algorithms to a matrix of pairwise distances estimated using the Tamura-Nei model, and then selecting the topology with superior log likelihood value. The percentage of replicate trees in which the associated taxa clustered together in the bootstrap test (1000 replicates) are shown next to the branches; bootstrap values < 90% were hidden

Eighteen EV-B types were identified in 141 samples, representing 40.2% of the NPEVs identified in this study. CVB4 (16.3%) was the most predominant EV-B serotype detected, followed by echovirus 7 (E7) (11.3%), CVB2 (9.9%), and E11 (9.9%) (Table 1). For most EV-B types, the VP1-encoding sequences found in this study were close to each other and were closely related to those of EVs previously reported: for instance, the CVB5 belonged to the genogroup A already described [24], the E20 were close to viruses previously sampled in Madagascar [25], while CVB2s, CVB3s, CVB4s and E29s were close to isolates reported in India in the 2000s [26–28]. By contrast, some isolates found in this study were not related to lineages already described in the corresponding types. For instance, the sole E19 isolate and the E2 and E12 isolates formed specific clusters that do not include viruses found elsewhere (Fig. 5). Similarly, the E24 isolates fell into two separate clusters that did not contain any other sequences.

**Fig. 6:**
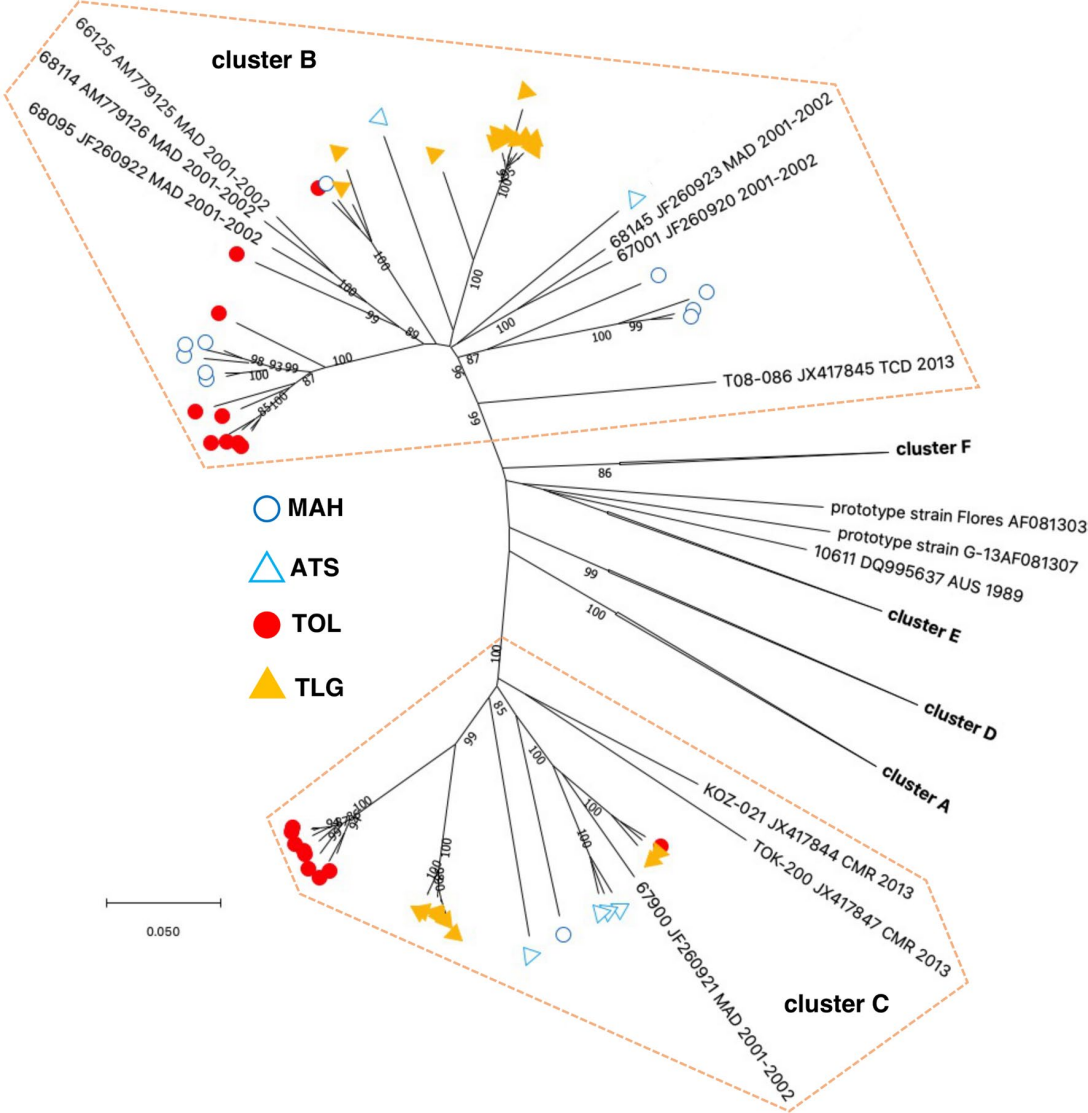
Phylogenetic relationships based on the VP1 sequences of CVA13s. For better legibility, some clusters were collapsed and the name of the isolate from this study are not indicated. Circles and triangles indicate the district where each isolate was sampled. For the other sequences, their respective GenBank accession number, country (ISO 3166-1 alpha-3 codes) and year of sampling are indicated, when known. The percentage of replicate trees in which the associated taxa clustered together in the bootstrap test (1000 replicates) are shown next to the branches; bootstrap values < 95% were hidden

Overall, 180 EV-C isolates were identified and accounted for 51.3% of the NPEVs. They were allocated in six types (CVA11, CVA13, CVA17, CVA20, CVA24, and EV-C99) that had already been reported in the island (Table 1). Members of the CVA13 type were the most abundant, accounting for most than one third of the EV-C. Previous studies based on the VP1-encoding sequence revealed a high genetic diversity within this type and at least 6 clusters of CVA13 VP1 sequences (A–F) have been reported [29, 30]. All sequences of this study fell into clusters B and C that contain strains previously isolated in Madagascar [31] and strains that circulated in central Africa in the 2000s and 2010 [29] (Fig. 6).

All EV-C99 fell into the previously described cluster C [32], which contains isolates sampled in Madagascar, in central Africa and in Europe in the 2000s (personal communication). In the VP1-encoding sequence, the CVA11, CVA17, CVA20 and CVA24 isolates were genetically close to EVs isolated in Madagascar in 2001–2002 [31].

**Fig. 7:**
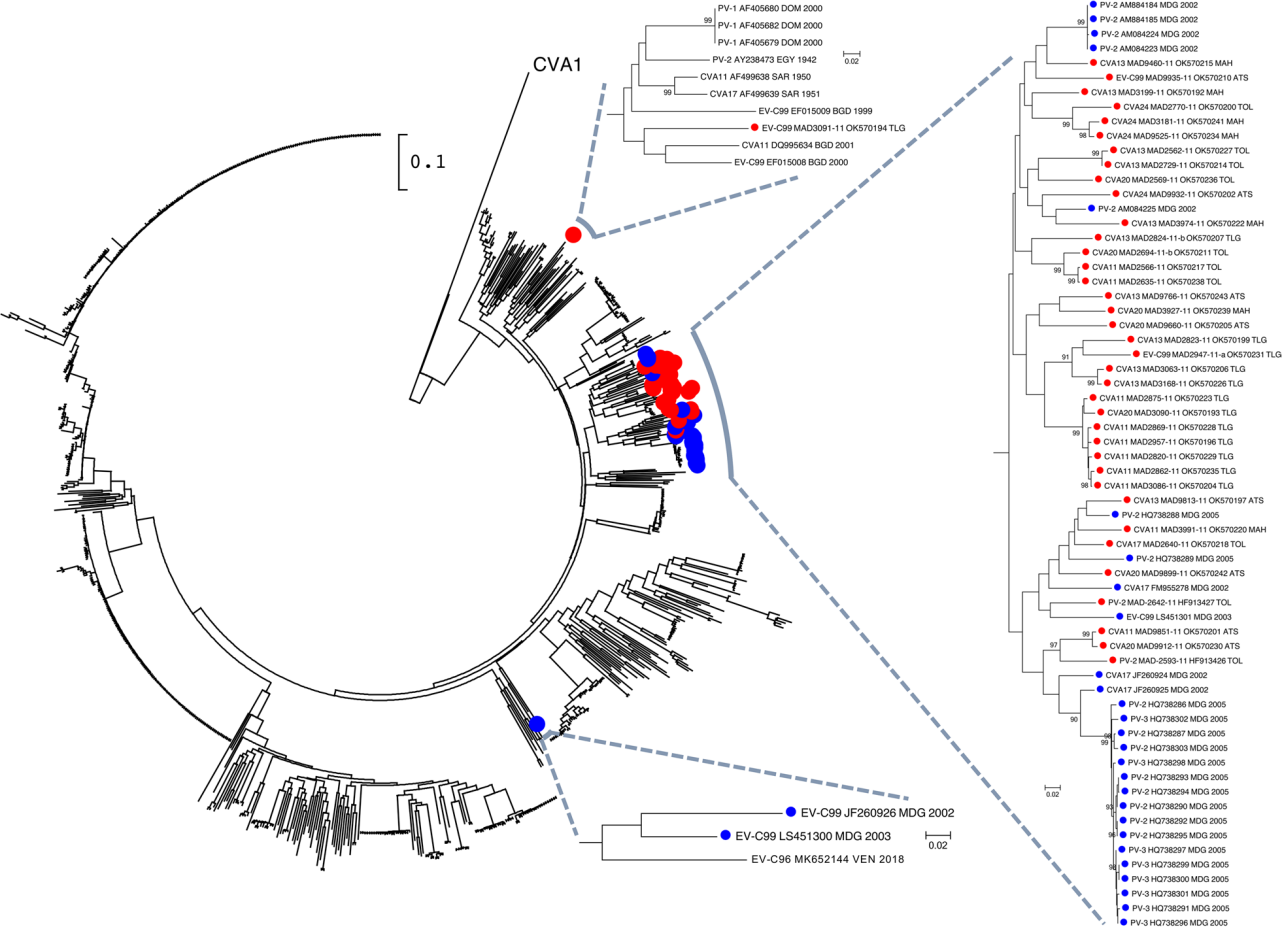
Phylogenetic relationships of EV-Cs, based on the full-length 3D-encoding region. The main tree contains all 3D-encoding sequences of EV-Cs belonging to cluster IV found in GenBank. The subtrees with Madagascan sequences are expanded. Red circles indicate isolates from this study (with the name of the corresponding district), blue circles indicate sequences sampled in Madagascar in previous studies (with their respective year of sampling). The other sequences are named by using their respective GenBank accession number, country (ISO 3166-1 alpha-3 codes) and year of sampling. The percentage of replicate trees in which the associated taxa clustered together in the bootstrap test (1000 replicates) are shown next to the branches if < 90%. The CVA1 sequence was used as outlier for rooting the tree

In order to study the genetic links between the EV-Cs isolated in this study and the different VDPVs that circulated in the island in the 2000s and 2010s [5–7], the genome of 52 EV-C representative of different lineages were sequenced. As usually observed in EV ecosystems, phylogenetic incongruences were observed between the trees based on the 5′UTR, the capsid-encoding region, and the non-structural part of the genome (Additional file 3: Figure S2); these incongruences are due to recombination events, known to be frequent amongst EVs [33]. It has been previously shown that the EV-C non-structural sequences fall into four phylogenetic clusters, each of which gathering viruses that can recombine one with another [34]. All the EV-C types found in this study belong to the cluster IV. To study the phylogenetic relationships between the Malagasy EV-C and EV-C circulating elsewhere, the 3D-encoding sequences of EV-C sampled in the island in this study or in previous ones were aligned with all the full-length 3D sequences of cluster IV EV-C sampled worldwide. In the corresponding phylogenetic tree, most of the Madagascan EV-C cluster together in a branch that does not contain any other EV-C from another country (Fig. 7). The three exceptions were one EV-C99 from this study (MAD3091-11) and two EV-C99 sampled in the 2000s, which fall in two separate branches and were not peculiarly close to any other sequences available in GenBank.

## Discussion

Previous studies have described the frequent presence of NPEVs in Madagascar and underlined the high frequency of EVs of species C [5, 25, 35]. Nonetheless, these studies only focused on the southern part of the island, where most VDPVs were detected. Nothing was known regarding putative differences between NPEV populations circulating in different parts of Madagascar. To determine whether peculiar treats of the NPEV population circulating in the southern part of Madagascar could explain why cVDPVs preferentially emerged in this region, we conducted a comparative study based on stools collected in the same period from healthy children living in two southern and two northern regions of Madagascar. This study was carried out in 2011, when next-generation sequencing was less affordable than nowadays. For this reason, we chose to detect the NPEVs through virus isolation rather than through direct molecular screening. Because it is known that RD cells are poorly sensitive to NPEVs of the species C [36, 37], we screened each sample on RD cells and HEp-2c cells, known to be more sensitive to EV-C [38]. The parallel use of these two cell lines is expected to allow the detection of most types of EV-A, -B, C and D, including the EV-C that commonly recombine with PV vaccine strains.

This study revealed an extensive circulation of NPEVs among healthy children, with an overall isolation rate of 26.8% (351/1309). This value is in the range of NPEV isolation rates already reported in healthy people, but the isolation range notably varies from study to study: 8.9% in children under 15 years old in the border area of China and Myanmar in 2009 [39], 24.7% in children under 6 years in the Philippines [40], 24.2% in children under 8 years old in Ghana [41], and 51% in people under 21 years old in Mongolia [42].

The molecular characterization of NPEVs showed that the EV-C species were the most prevalent followed by EV-B species and in the last the EV-A species. No EV-D species were detected during our investigation. Since members of this species generally produce clear CPE when grown in RD and L20B cells [11], it can be assumed that EV-D were not actively circulating when the study was conducted.

Interestingly, some VP1-encoding sequences fell in phylogenetic clusters that exclusively contain sequences of viruses isolated in Madagascar. These findings could be due to Madagascar geographic isolation, which could favor the appearance of topotypes in absence of the recent introduction of strains from other countries. Nonetheless, it is also possible that the genetic data available in public databases provides a biased overview of the actual area of circulation of some EV types and lineages. Indeed, only a few sequences are available for some EV types, such as for instance the types E2 or E12. It is to noted that CVA7 was the most prevalent EV-A type detected in this study although this type is rarely reported nowadays [43], although it was widely detected in the 1950s and 1960s during outbreaks of flaccid paralysis [44, 45].

The fact that most of the non-structural sequences from EV-C sampled in Madagascar in the 2000s and in 2011 cluster together in a branch that does not contain any sequences from viruses sampled in other countries suggests that EV-C mainly evolved during this period without massive importation of strains from other geographic regions. Whether it means that EV-C importations are rare in Madagascar or whether foreign strains are outcompeted by the indigenous ones remains to be determined.

No differences regarding the types that circulate in the northern and the southern districts was noted. Importantly, the usual partners of the PV vaccine strains (CVA11, CVA13, CVA17 and CVA20) were found both in the North and in the South. Inside each type, no obvious clustering based on the origin (North vs South of the island) was observed, which indicates the probable co-circulation of different lineages through the island during the same year. By contrast, the northern and southern districts differed by two important features: first, the isolation rate was higher in the South; second, the relative abundance of EV-B and EV-C greatly differs between the North and the South. The donors were randomly selected in all sites, which is expected to limit sampling bias. As all the samples were processed using the same protocols in the same lab, technical biases can be ruled out. Because this study was conducted on a short period of time, it is impossible to determine whether this huge discrepancy is constant over the time. Nonetheless, a previous study conducted 10 years before in the district of Taolagnaro also showed a predominance of EV-C among circulating NPEVs since they accounted for two-thirds of the NPEVs detected by using both RD and HEp-2c cell lines [35]. The climate could be one of the factors that explain the difference observed between the northern districts and the southern one. Madagascar has a subtropical climate with a warm rainy season (summer, from November to March) and a cooler dry season (winter, from April through October). There are huge differences between the North and the South in the island: the south is an arid region and the district of Toliara is the driest zone of the island.

The reasons that explain this discrepancy remain to be deciphered but the fact that EV-C were majority in regions where cVDPVs repeatedly emerged is striking. The unknown factors responsible for the high frequency of EV-Cs in this region could also favor the circulation of vaccine PV strains. Furthermore, the high frequency of EV-C could facilitate the recombination of vaccine PV strains with NPEV partners, particularly CVA13, 17 or 20. Indeed, the production of recombinant genomes requires co-infections, which are probably more likely with majority types. It is known that most VDPVs feature recombinant genomes and it has been shown that NPEV sequences play an important role in the VDPV neurovirulence level [46–48]. Therefore, environmental surveillance constitutes a precious tool in the GPEI strategy as it enables the early detection of the circulation of Sabin strains but also provides clues for the presence in the same area of non-polio EV-Cs that frequently recombine with them.

## Conclusions

This study demonstrated that the exposure to NPEVs is not uniform in Madagascar. Coupled with the low immunity against poliovirus, the particular enteroviral ecosystem that exists in the South of Madagascar could be a favorable factor for increasing the emergence risk of recombinant VDPVs in this region.

## Supplementary Information

**Additional file 1: Table S1.** Accession numbers of the sequences used to draw the tree displayed in Fig. 7.

**Additional file 2: Figure S1.** Detection of NPEVs on RD and HEp-2c cells.

**Additional file 3: Figure S2.** Phylogenetic trees of Madagascan EV-Cs based on the 5′UTR, the VP1- and the 3D-encoding sequences. The isolates are colour-coded according to their respective type; triangles indicate isolates from this study, circles isolates from previous works.

## Data Availability

The datasets generated and/or analyzed during the current study are available in the GenBank repository (Accession numbers: KJ768669-KJ768838, KR232691-KR232798 and OK570192-OK570243).
