# Supplementary material for: Enterovirus detection in different regions of Madagascar reveals a higher abundance of enteroviruses of species C in areas where several outbreaks of vaccine-derived polioviruses occurred
Source: BMC Infect Dis. 2022 Nov 8;22:821. doi: 10.1186/s12879-022-07826-0 (PMC9641760; doi:10.1186/s12879-022-07826-0)
Supplement: Supplementary file 1 — Additional file 1: Table S1. Accession numbers of the sequences used to draw the tree displayed in Fig. 7. [file 12879_2022_7826_MOESM1_ESM.pdf]

Table S1. Accession numbers of the sequences used to draw the tree displayed in Figure 7.

Sequences retrieved from GenBank	Sequences from this study
CVA1 JX174176	CVA11 MAD2566-11 OK570217 TOL
AB180070	CVA11 MAD2635-11 OK570238 TOL
AB180071	CVA11 MAD2820-11 OK570229 TLG
AB180072	CVA11 MAD2862-11 OK570235 TLG
AB180073	CVA11 MAD2869-11 OK570228 TLG
AB205395	CVA11 MAD2875-11 OK570223 TLG
AB769152	CVA11 MAD2957-11 OK570196 TLG
AB769154	CVA11 MAD3086-11 OK570204 TLG
AB769156	CVA11 MAD3991-11 OK570220 MAH
AB769159	CVA11 MAD9851-11 OK570201 ATS
AB769160	CVA13 MAD2562-11 OK570227 TOL
AB769161	CVA13 MAD2729-11 OK570214 TOL
AB769162	CVA13 MAD2823-11 OK570199 TLG
AB769163	CVA13 MAD2824-11-b OK570207 TLG
AB769164	CVA13 MAD3063-11 OK570206 TLG
AB769165	CVA13 MAD3168-11 OK570226 TLG
AF111953	CVA13 MAD3199-11 OK570192 MAH
AF111961	CVA13 MAD3974-11 OK570222 MAH
AF111966	CVA13 MAD9460-11 OK570215 MAH
AF111981	CVA13 MAD9766-11 OK570243 ATS
AF111982	CVA13 MAD9813-11 OK570197 ATS
AF111983	CVA17 MAD2640-11 OK570218 TOL
AF111984	CVA20 MAD2569-11 OK570236 TOL
AF405662	CVA20 MAD2694-11-b OK570211 TOL
AF405663	CVA20 MAD3090-11 OK570193 TLG
AF405664	CVA20 MAD3927-11 OK570239 MAH
AF405665	CVA20 MAD9660-11 OK570205 ATS
AF405666	CVA20 MAD9899-11 OK570242 ATS
AF405667	CVA20 MAD9912-11 OK570230 ATS
AF405668	CVA24 MAD2770-11 OK570200 TOL
AF405669	CVA24 MAD3181-11 OK570241 MAH
AF405670	CVA24 MAD9525-11 OK570234 MAH
AF405671	CVA24 MAD9932-11 OK570202 ATS
AF405672	EV-C99 MAD2947-11-a OK570231 TLG
AF405673	EV-C99 MAD3091-11 OK570194 TLG
AF405674	EV-C99 MAD9935-11 OK570210 ATS
AF405675	CVA11 MAD2862-11 OK570235 TLG
AF405676	CVA11 MAD2869-11 OK570228 TLG
AF405677	CVA11 MAD2875-11 OK570223 TLG
AF405678	CVA11 MAD2957-11 OK570196 TLG
AF405679	CVA11 MAD3086-11 OK570204 TLG

AF405680	CVA11 MAD3991-11 OK570220 MAH
AF405681	CVA11 MAD9851-11 OK570201 ATS
AF405682	CVA13 MAD2562-11 OK570227 TOL
AF405683	CVA13 MAD2729-11 OK570214 TOL
AF405684	CVA13 MAD2823-11 OK570199 TLG
AF405685	CVA13 MAD2824-11-b OK570207 TLG
AF405686	CVA13 MAD3063-11 OK570206 TLG
AF405687	CVA13 MAD3168-11 OK570226 TLG
AF405688	CVA13 MAD3199-11 OK570192 MAH
AF405689	CVA13 MAD3974-11 OK570222 MAH
AF405690	CVA13 MAD9460-11 OK570215 MAH
AF416342	CVA13 MAD9766-11 OK570243 ATS
AF448782	CVA13 MAD9813-11 OK570197 ATS
AF448783	CVA17 MAD2640-11 OK570218 TOL
AF458333	CVA20 MAD2569-11 OK570236 TOL
AF462418	CVA20 MAD2694-11-b OK570211 TOL
AF462419	CVA20 MAD3090-11 OK570193 TLG
AF465511	CVA20 MAD3927-11 OK570239 MAH
AF465515	CVA20 MAD9660-11 OK570205 ATS
AF499636	CVA20 MAD9899-11 OK570242 ATS
AF499638	CVA20 MAD9912-11 OK570230 ATS
AF499639	CVA24 MAD2770-11 OK570200 TOL
AF499640	CVA24 MAD3181-11 OK570241 MAH
AF499642	CVA24 MAD9525-11 OK570234 MAH
AF538840	CVA24 MAD9932-11 OK570202 ATS
AF538841	EV-C99 MAD2947-11-a OK570231 TLG
AF538842	EV-C99 MAD3091-11 OK570194 TLG
AF538843	EV-C99 MAD9935-11 OK570210 ATS
AF541919	CVA11 MAD2566-11 OK570217 TOL
AF546702	CVA11 MAD2635-11 OK570238 TOL
AJ132960	CVA11 MAD2820-11 OK570229 TLG
AJ132961	CVA11 MAD2862-11 OK570235 TLG
AJ416942	CVA11 MAD2869-11 OK570228 TLG
AJ430385	CVA11 MAD2875-11 OK570223 TLG
AJ544513	CVA11 MAD2957-11 OK570196 TLG
AM040035	CVA11 MAD3086-11 OK570204 TLG
AM040036	CVA11 MAD3991-11 OK570220 MAH
AM040037	CVA11 MAD9851-11 OK570201 ATS
AM040038	CVA13 MAD2562-11 OK570227 TOL
AM040039	CVA13 MAD2729-11 OK570214 TOL
AM084223	CVA13 MAD2823-11 OK570199 TLG
AM084224	CVA13 MAD2824-11-b OK570207 TLG
AM084225	CVA13 MAD3063-11 OK570206 TLG
AM884184	CVA13 MAD3168-11 OK570226 TLG
AM884185	CVA13 MAD3199-11 OK570192 MAH
AY184219	CVA13 MAD3974-11 OK570222 MAH

AY184221	CVA13 MAD9460-11 OK570215 MAH
AY238473	CVA13 MAD9766-11 OK570243 ATS
AY278550	CVA13 MAD9813-11 OK570197 ATS
AY278551	CVA17 MAD2640-11 OK570218 TOL
AY278552	CVA20 MAD2569-11 OK570236 TOL
AY278553	CVA20 MAD2694-11-b OK570211 TOL
AY421739	CVA20 MAD3090-11 OK570193 TLG
AY560657	CVA20 MAD3927-11 OK570239 MAH
AY876912	CVA20 MAD9660-11 OK570205 ATS
AY876913	CVA20 MAD9899-11 OK570242 ATS
AY928383	CVA20 MAD9912-11 OK570230 ATS
AY928384	CVA24 MAD2770-11 OK570200 TOL
AY928385	CVA24 MAD3181-11 OK570241 MAH
AY928386	CVA24 MAD9525-11 OK570234 MAH
AY928387	CVA24 MAD9932-11 OK570202 ATS
AY948201	EV-C99 MAD2947-11-a OK570231 TLG
MT432082	EV-C99 MAD3091-11 OK570194 TLG
MT432083	EV-C99 MAD9935-11 OK570210 ATS
MT432084	
MT432085	
MT432086	
MT432087	
MT432088	
MT432089	
MT432090	
MT432091	
MT432092	
MT432093	
MT432094	
MT432154	
MT432095	
MT432098	
MT432099	
MT432100	
MT432101	
MT432102	
MT432103	
MT432104	
MT432105	
MT432119	
MT432120	
MT432121	
MT432122	
MT432123	
MT432124	
MT432125	

MT432126	
MT432127	
MT432131	
MT432132	
MT432133	
MT432135	
CXA21CG	
CXA24CG	
DQ443001	
DQ443002	
DQ890385	
DQ890386	
DQ890387	
DQ890388	
DQ995633	
DQ995634	
DQ995635	
DQ995636	
DQ995637	
DQ995638	
DQ995639	
DQ995640	
DQ995641	
DQ995642	
DQ995643	
DQ995644	
DQ995645	
DQ995646	
EF015008	
EF015009	
EF015010	
EF015011	
EF015012	
EF015013	
EF015014	
EF015015	
EF015016	
EF015017	
EF015018	
EF015019	
EF015020	
EF015021	
EF015022	
EF015023	
EF015024	
EF015025	

EF015026	
EF015027	
EF015028	
EF015029	
EF015030	
EF015031	
EF015032	
EF015033	
EF015034	
EF015035	
EF015036	
EF015037	
EF015038	
EF015039	
EF015040	
EF026081	
EF456706	
EF555644	
EF555645	
EF682343	
EF682344	
EF682345	
EF682346	
EF682347	
EF682348	
EF682349	
EF682350	
EF682351	
EF682352	
EF682353	
EF682354	
EF682355	
EF682356	
EF682357	
EF682358	
EF682359	
MT432146	
MT432140	
MT432142	
MT432143	
MT432149	
MT432150	
EU566934	
EU566935	
EU566936	
EU566937	

EU566938	
EU566939	
EU566940	
EU566941	
EU566942	
EU566943	
EU566944	
EU566945	
EU566946	
EU566947	
EU566948	
EU566949	
EU566950	
EU684056	
EU684057	
EU794953	
EU794954	
EU794955	
EU794956	
EU794957	
EU794958	
EU794959	
EU794960	
EU794961	
EU794962	
EU794963	
EU794964	
FJ460223	
FJ460224	
FJ460225	
FJ460226	
FJ517649	
FJ751915	
FJ769378	
FJ769379	
FJ769380	
FJ769381	
FJ769382	
FJ769383	
FJ769384	
FJ769385	
FJ842158	
FJ842159	
FJ859058	
FJ859059	
FJ859060	

FJ859061	
FJ859062	
FJ859063	
FJ859064	
FJ859183	
FJ859184	
FJ859185	
FJ859186	
FJ859187	
FJ859188	
FJ859189	
FJ859190	
FJ859191	
FJ898290	
FJ914252	
FM955278	
GQ984141	
GU180608	
GU256222	
GU390707	
HF913426	
HF913427	
HM107832	
HM107833	
HM107834	
HQ415758	
HQ415759	
HQ738286	
HQ738287	
HQ738288	
HQ738289	
HQ738290	
HQ738291	
HQ738292	
HQ738293	
HQ738294	
HQ738295	
HQ738296	
HQ738297	
HQ738298	
HQ738299	
HQ738300	
HQ738301	
HQ738302	
HQ738303	
JF260924	

JF260925	
JF260926	
JF742576	
JF742577	
JF742578	
JF742579	
JF838278	
JN228097	
JX274980	
JX274981	
JX274982	
JX274983	
JX274984	
JX274985	
JX274987	
JX274993	
JX274995	
JX274999	
JX275008	
JX275015	
JX275032	
JX275071	
JX275140	
JX275147	
JX275162	
JX275184	
JX275238	
JX275266	
JX275352	
JX569709	
JX569710	
JX569711	
JX569712	
KC784367	
KC784368	
KC784372	
KC880365	
KC880366	
KC880367	
KC880368	
KC880369	
KC880370	
KC880371	
KC880372	
KC880373	
KC880374	

KC880375	
KC880376	
KC880377	
KC880378	
KC880379	
KC880380	
KC880381	
KC880382	
KF128998	
KF129411	
KF129412	
KF495604	
KF537633	
KF667358	
KF667359	
KF667360	
KF667361	
KF725085	
KJ019831	
KJ019832	
KJ019833	
KJ155495	
KJ155496	
KJ155499	
KJ155500	
KJ170436	
KJ170437	
KJ170438	
KJ170439	
KJ170440	
KJ170441	
KJ170442	
KJ170443	
KJ170444	
KJ170445	
KJ170446	
KJ170447	
KJ170448	
KJ170449	
KJ170450	
KJ170451	
KJ170452	
KJ170453	
KJ170454	
KJ170455	
KJ170456	

KJ170457	
KJ170458	
KJ170459	
KJ170460	
KJ170461	
KJ170462	
KJ170463	
KJ170464	
KJ170465	
KJ170466	
KJ170467	
KJ170468	
KJ170469	
KJ170470	
KJ170471	
KJ170472	
KJ170473	
KJ170474	
KJ170475	
KJ170476	
KJ170477	
KJ170478	
KJ170479	
KJ170480	
KJ170481	
KJ170482	
KJ170483	
KJ170484	
KJ170485	
KJ170486	
KJ170487	
KJ170488	
KJ170489	
KJ170490	
KJ170491	
KJ170492	
KJ170493	
KJ170494	
KJ170495	
KJ170496	
KJ170497	
KJ170498	
KJ170499	
KJ170500	
KJ170501	
KJ170502	

KJ170503	
KJ170504	
KJ170505	
KJ170506	
KJ170507	
KJ170508	
KJ170509	
KJ170510	
KJ170511	
KJ170512	
KJ170513	
KJ170514	
KJ170515	
KJ170516	
KJ170517	
KJ170518	
KJ170519	
KJ170520	
KJ170521	
KJ170522	
KJ170523	
KJ170524	
KJ170525	
KJ170526	
KJ170527	
KJ170528	
KJ170529	
KJ170530	
KJ170531	
KJ170532	
KJ170576	
KJ170577	
KJ170578	
KJ170579	
KJ170580	
KJ170581	
KJ170582	
KJ170583	
KJ170584	
KJ170585	
KJ170586	
KJ170587	
KJ170588	
KJ170589	
KJ170590	
KJ170591	

KJ170592	
KJ170593	
KJ170594	
KJ170595	
KJ170596	
KJ170597	
KJ170598	
KJ170599	
KJ170600	
KJ170601	
KJ170602	
KJ170603	
KJ170604	
KJ170605	
KJ170606	
KJ170607	
KJ170608	
KJ170609	
KJ170610	
KJ170611	
KJ170612	
KJ170613	
KJ170614	
KJ170615	
KJ170616	
KJ170617	
KJ170618	
KJ170619	
KJ170620	
KJ170621	
KJ170622	
KJ170623	
KJ170624	
KJ170625	
KJ170626	
KJ170627	
KJ170628	
KJ170629	
KJ170630	
KJ170631	
KJ170632	
KJ170633	
KJ170634	
KJ170635	
KJ170636	
KJ170637	

KJ170638	
KJ170639	
KJ170640	
KJ170641	
KJ170642	
KJ170643	
KJ170644	
KJ170645	
KJ170646	
KJ170647	
KJ170648	
KJ170649	
KJ170650	
KJ170651	
KJ170652	
KJ170653	
KJ170654	
KJ170655	
KJ170656	
KJ170657	
KJ170658	
KJ170659	
KJ170660	
KJ170661	
KJ170662	
KJ170663	
KJ170664	
KJ170665	
KJ170666	
KJ170667	
KJ170668	
KJ170669	
KJ170670	
KJ170671	
KJ170672	
KJ170673	
KJ170674	
KJ170675	
KJ170676	
KJ170677	
KJ419273	
KJ419274	
KJ419275	
KJ419276	
KJ419277	
KJ857507	

KJ857508	
KM273014	
KP196613	
KP984753	
KP984754	
KR259355	
KR259358	
KR399988	
KR478685	
KR919804	
KT161266	
KT353719	
KT946713	
KT946715	
KT946716	
KT946717	
KU161395	
KU161396	
KU161397	
KU161398	
KU161399	
KU183495	
KU372652	
KU866422	
KX162678	
KX162679	
KX162680	
KX162681	
KX162682	
KX162683	
KX162684	
KX162685	
KX162686	
KX162687	
KX162688	
KX162689	
KX162690	
KX162691	
KX162692	
KX162693	
KX162694	
KX162695	
KX162696	
KX162697	
KX162698	
KX162699	

KX162700	
KX162701	
KX162702	
KX162703	
KX162704	
KX162705	
KX162706	
KX162708	
KX162710	
KX162711	
KX162712	
KX162713	
KX162714	
KX162715	
KX162716	
KX384961	
KX384962	
KX384963	
KX384964	
KY271947	
KY284011	
KY703697	
KY941931	
KY941932	
KY941933	
KY941934	
KY941935	
LC279542	
LR796218	
LS451300	
LS451301	
LT934387	
MF189567	
MF346171	
MF419263	
MF541373	
MF990304	
MF990307	
MG212427	
MG212428	
MG212429	
MG212430	
MG212431	
MG212432	
MG212433	
MG212434	

MG212435	
MG212436	
MG212437	
MG212438	
MG212439	
MG212440	
MG212441	
MG212442	
MG212443	
MG212444	
MG212445	
MG212446	
MG212447	
MG212448	
MG212449	
MG212450	
MG212451	
MG212452	
MG212453	
MG212454	
MG212455	
MG212456	
MG212457	
MG212458	
MG212459	
MG212460	
MG212461	
MG212462	
MG212463	
MG212464	
MG212465	
MG212466	
MG212467	
MG212468	
MG212469	
MG212471	
MG212472	
MG212474	
MG212475	
MG212476	
MG212477	
MG212478	
MG212479	
MG212480	
MG212481	
MG212482	

MG212483	
MG212484	
MG212486	
MG212487	
MG212489	
MG212490	
MG212491	
MG212493	
MG212494	
MG212495	
MG451807	
MG557561	
MG557562	
MG557563	
MG571840	
MG571841	
MG571844	
MG571860	
MG880745	
MG880747	
MG880748	
MG880749	
MG880750	
MG880751	
MG880752	
MG982663	
MH144606	
MH484164	
MH484166	
MH750912	
MH785183	
MK028134	
MK512667	
MK512668	
MK652144	
MK689071	
MK989717	
MK989718	
MK989719	
MK989720	
MK989721	
MK989722	
MN149910	
MN914197	
MN914201	
MN914202	

MN914203	
MN914204	
MN914205	
MN914206	
MN918613	
MT432082	
MT432083	
MT432084	
MT432085	
MT432086	
MT432087	
MT432088	
MT432089	
MT432090	
MT432091	
MT432092	
MT432093	
MT432094	
MT432095	
MT432096	
MT432097	
MT432098	
MT432099	
MT432100	
MT432101	
MT432102	
MT432103	
MT432104	
MT432105	
MT432106	
MT432107	
MT432108	
MT432109	
MT432110	
MT432111	
MT432112	
MT432113	
MT432114	
MT432115	
MT432119	
MT432120	
MT432121	
MT432122	
MT432123	
MT432124	
MT432125	

MT432126	
MT432127	
MT432128	
MT432129	
MT432130	
MT432131	
MT432132	
MT432133	
MT432134	
MT432135	
MT432137	
MT432138	
MT432139	
MT432140	
MT432141	
MT432142	
MT432143	
MT432144	
MT432145	
MT432146	
MT432147	
MT432148	
MT432149	
MT432150	
MT432151	
MT645947	
MT645948	
MT645949	
MT645950	
MT645951	
MW366963	
MW366964	
MW366965	
MZ396299	
MZ546188	
