# Supplementary figures and images for: Enterovirus detection in different regions of Madagascar reveals a higher abundance of enteroviruses of species C in areas where several outbreaks of vaccine-derived polioviruses occurred
Source: BMC Infect Dis. 2022 Nov 8;22:821. doi: 10.1186/s12879-022-07826-0 (PMC9641760; doi:10.1186/s12879-022-07826-0)

Figure S1. Detection of NPEVs on RD and HEp-2c cells.

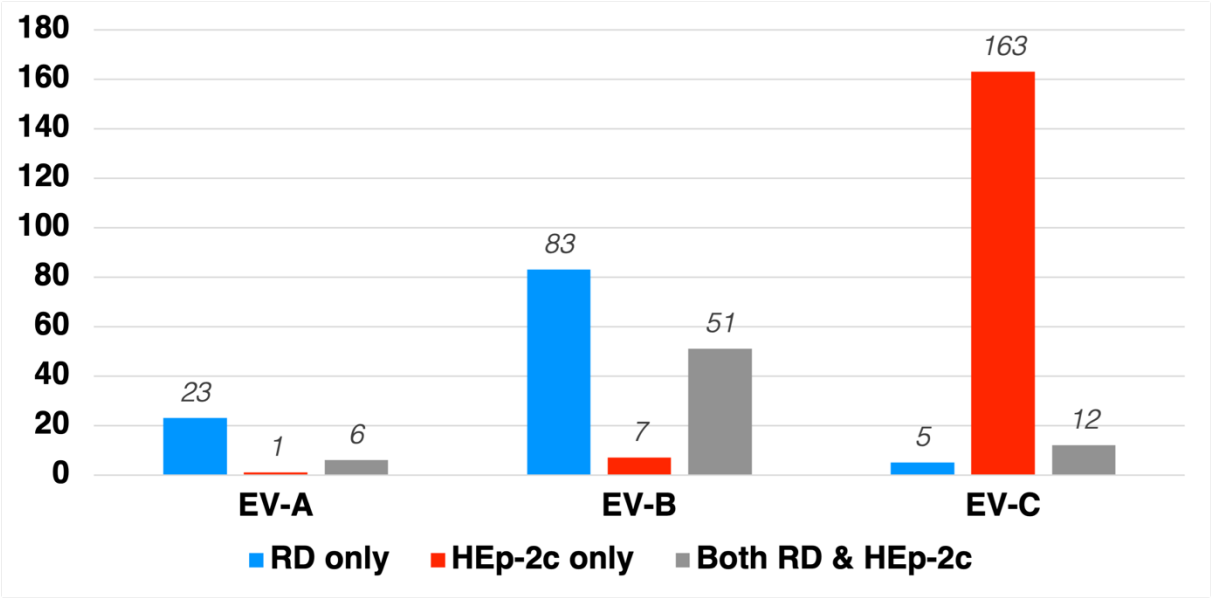

Supplement: Supplementary file 2 — Additional file 2: Figure S1. Detection of NPEVs on RD and HEp-2c cells. [file 12879_2022_7826_MOESM2_ESM.pdf]
