# Supplementary material for: Enterovirus detection in different regions of Madagascar reveals a higher abundance of enteroviruses of species C in areas where several outbreaks of vaccine-derived polioviruses occurred
Source: BMC Infect Dis. 2022 Nov 8;22:821. doi: 10.1186/s12879-022-07826-0 (PMC9641760; doi:10.1186/s12879-022-07826-0)

Figure S2. Phylogenetic trees of Madagascan EV-Cs based on the 5′UTR, the VP1- and the 3D-encoding sequences. The isolates are colour-coded according to their respective type; triangles indicate isolates from this study, circles isolates from previous works.

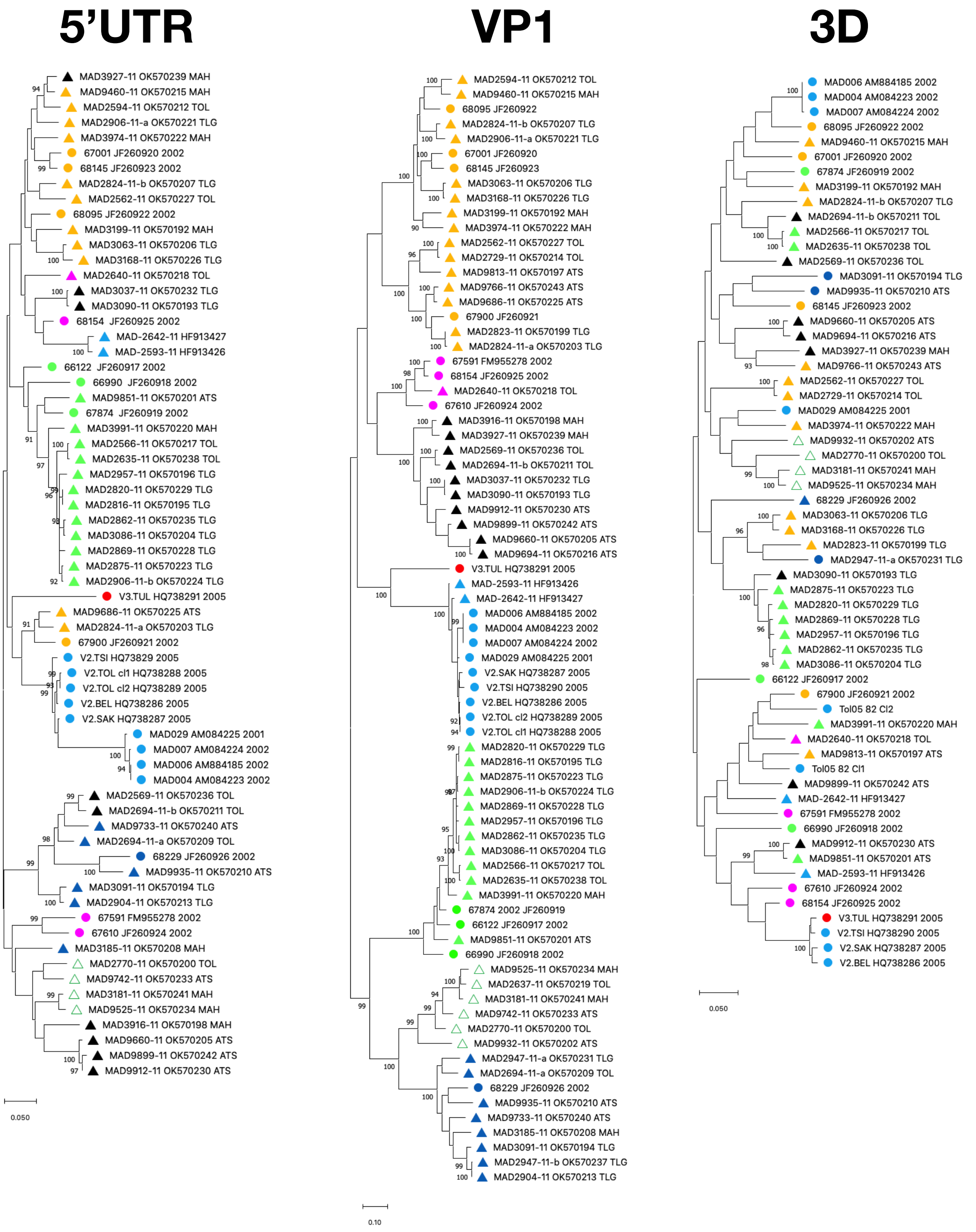

Supplement: Supplementary file 3 — Additional file 3: Figure S2. Phylogenetic trees of Madagascan EV-Cs based on the 5′UTR, the VP1- and the 3D-encoding sequences. The isolates are colour-coded according to their respective type; triangles indicate isolates from this study, circles isolates from previous works. [file 12879_2022_7826_MOESM3_ESM.pdf]
